# NS4A protein as a marker of HCV history suggests that different HCV genotypes originally evolved from genotype 1b

**DOI:** 10.1186/1743-422X-8-317

**Published:** 2011-06-23

**Authors:** Muhammad T Sarwar, Humera Kausar, Bushra Ijaz, Waqar Ahmad, Muhammad Ansar, Aleena Sumrin, Usman A Ashfaq, Sultan Asad, Sana Gull, Imran Shahid, Sajida Hassan

**Affiliations:** 1 Centre of Excellence in Molecular Biology, University of the Punjab, Lahore-53700, Pakistan

## Abstract

**Background:**

The 9.6 kb long RNA genome of Hepatitis C virus (HCV) is under the control of RNA dependent RNA polymerase, an error-prone enzyme, for its transcription and replication. A high rate of mutation has been found to be associated with RNA viruses like HCV. Based on genetic variability, HCV has been classified into 6 different major genotypes and 11 different subtypes. However this classification system does not provide significant information about the origin of the virus, primarily due to high mutation rate at nucleotide level. HCV genome codes for a single polyprotein of about 3011 amino acids which is processed into structural and non-structural proteins inside host cell by viral and cellular proteases.

**Results:**

We have identified a conserved NS4A protein sequence for HCV genotype 3a reported from four different continents of the world i.e. Europe, America, Australia and Asia. We investigated 346 sequences and compared amino acid composition of NS4A protein of different HCV genotypes through Multiple Sequence Alignment and observed amino acid substitutions C_22_, V_29_, V_30_, V_38_, Q_46 _and Q_47 _in NS4A protein of genotype 1b. Furthermore, we observed C_22 _and V_30 _as more consistent members of NS4A protein of genotype 1a. Similarly Q_46 _and Q_47 _in genotype 5, V_29_, V_30_, Q_46 _and Q_47 _in genotype 4, C_22_, Q_46 _and Q_47 _in genotype 6, C_22_, V_38_, Q_46 _and Q_47 _in genotype 3 and C_22 _in genotype 2 as more consistent members of NS4A protein of these genotypes. So the different amino acids that were introduced as substitutions in NS4A protein of genotype 1 subtype 1b have been retained as consistent members of the NS4A protein of other known genotypes.

**Conclusion:**

These observations indicate that NS4A protein of different HCV genotypes originally evolved from NS4A protein of genotype 1 subtype 1b, which in turn indicate that HCV genotype 1 subtype 1b established itself earlier in human population and all other known genotypes evolved later as a result of mutations in HCV genotype 1b. These results were further confirmed through phylogenetic analysis by constructing phylogenetic tree using NS4A protein as a phylogenetic marker.

## Introduction

Hepatitis C virus belongs to Flaviviridae family of viruses and its chronic infection has affected 350 million people worldwide [1]. HCV has a positive-sense single-stranded RNA genome of about 9.6 kb that has one single open reading frame and conserved un-translated regions (UTRs) at the 5' and 3' ends [2]. Within host cell the polyprotein is processed into structural (Core, E1, E2 and P7) and nonstructural proteins (NS2, NS3, NS4A, NS4B, NS5A and NS5B). Nonstructural 5B (NS5B) protein is an RNA-dependent RNA polymerase that is responsible for viral genome replication [3]. The error-prone nature of this enzyme is responsible for a high mutation rate in HCV. Based on nucleotide sequence comparison analysis in 5'UTR, Core/E1 and NS5B regions six major HCV genotypes (HCV-1 to HCV-6) have been described, each containing multiple subtypes (e.g., 1a, 1b, 1c etc). In terms of genetic variability, genotypes differ from each other by 31 to 33% and subtype by 20 to 25% [4].

Though HCV classification system has evolved considerably [5,6], it does not provide convincing information about origin of the virus. Suzuki and Nei used amino acid sequences of hemagglutinin genes instead of nucleotide sequences in their work on origin and evolution of influenza virus and they reported that amino acid sequences provide more reliable information in establishing evolutionary relationship than nucleotide sequences when the sequence divergence is high [7]. During our protein blast analysis http://blast.ncbi.nlm.nih.gov/Blast.cgi?PAGE=Proteins of NS4A gene (HCV genotype 3a) isolated from Pakistani population, we observed a relatively conserved nature of NS4A protein. Furthermore, we observed occasional amino acid substitutions in the NS4A protein sequences from genotype 3a.

NS4A protein is a small protein consisting total of 54 amino acids and it functions as cofactor of NS3 protease in viral life cycle. NS3-4A serine protease is a non-covalent, heterodimer complex formed by the association of two proteins, the N-terminal serine protease domain of NS3 (catalytic subunit) and NS4A cofactor (activation subunit). NS3-4A serine protease has a role in HCV polyprotein processing and is responsible for proteolytic cleavage at NS3/NS4A, NS4A/NS4B, NS4B/NS5A and NS5A/NS5B junctions to release individual proteins from the polyprotein [8-18].

The purpose of this study is to establish the identity of the parent HCV genotype that first established itself in human population. We have analyzed amino acid sequences of NS4A protein of all known Hepatitis C virus genotypes through Multiple Sequence Alignment and by constructing a phylogenetic tree using CLC sequence viewer software. We used NS4A protein due to many reasons. First of all due to its relatively conserved nature, second the occasional amino acid substitutions that we observed and third due to availability of large number of sequences for this region in sequence databases from all over the world. We have used amino acid substitutions as a tool because it would be logical to think that when an amino acid substitution is introduced into NS4A protein it will be retained in future progenies until mutated again. Due to a relatively conserved nature of NS4A protein, some of these amino acid substitutions might travel a long distance across different HCV genotypes as HCV evolved. If we follow such substitutions across different HCV genotypes it can provide valuable information about evolution of NS4A protein, and in turn about evolution of HCV. Phylogenetic tree was constructed using UPGMA (Unweighted Pair Group Method with Arithmetic Mean) method to support our results.

## Material and methods

Total of 346 nucleotide sequences were randomly selected and downloaded from Hepatitis C Virus Database http://www.hcvdb.org and GenBank http://www.ncbi.nlm.nih.gov representing 6 different HCV genotypes. The 346 sequences included in this study were reported from all over the world; France, Germany, UK, Switzerland, Ireland, Belgium, Spain, Portugal, Denmark, Sweden, Russia, Japan, China, Korea, Indonesia, Hong Kong, Thailand, Viet Nam, Pakistan, Singapore, India, Australia, USA, Canada, Algeria, Egypt, Cameroon and South Africa representing Europe, Asia, North America and Africa (Table 1). These nucleotide sequences were then adjusted for NS4A gene region using BioEdit software http://www.mbio.ncsu.edu/bioedit/bioedit.html and isolated H77 as a reference sequence http://www.hcvdb.org/gene_detail.asp?gene_id=64592. Amino acid sequences were deduced for these sequences using EXPASY protein translate tool http://expasy.org/tools/dna.html. The amino acid sequences were then fed to CLC sequence viewer 6 http://www.clcbio.com/index.php?id=28 for Multiple Sequence Alignment (MSA) to be performed. CLC sequence a viewer 6 is freely available software.

**Table 1:** Number of amino acid sequences of NS4A protein from different countries used in this study

S/N	Country names	Number of sequences used from individual genotypes								Total
			
		G-1b	G-1a	G-1c	G-2	G-3	G-4	G-5	G-6	
1	France	16	-	-	-	10	3	15	1	45
2	Germany	4	-	-	-	2	-	-	-	6
3	UK	-	12	-	1	15	2	1	2	33
4	Switzerland	6	11	-	-	4	-	-	-	21
5	Ireland	9	-	-	-	-	-	-	-	9
6	Belgium	-	-	-	-	1	-	8	-	9
7	Spain	-	-	-	-	-	1	1	-	2
8	Portugal	-	-	-	-	-	2	-	-	2
9	Denmark	-	-	-	-	1	-	-	-	1
10	Sweden	1	-	-	-	-	-	-	-	1
11	Russia	1	-	-	-	-	-	-	-	1
12	Japan	13	2	-	34	3	-	-	4	56
13	China	3	-	-	-	-	-	-	2	5
14	Korea	1	-	-	-	-	-	-	-	1
15	Indonesia	-	-	1	5	1	2	-	-	9
16	Hong Kong	-	-	-	-	-	-	-	14	14
17	Thailand	-	-	-	-	1	-	-	6	7
18	Viet Nam	-	-	-	1	-	-	-	1	2
19	Pakistan	-	-	-	-	7	-	-	-	7
20	Singapore	-	6	-	-	1	-	-	-	7
21	India	-	-	2	-	1	-	-	-	3
22	Australia	1	7	-	-	5	-	-	-	13
23	USA	17	9	-	16	6	12	1	-	61
24	Canada	-	7	-	-	-	-	-	-	7
25	Algeria	-	-	-	-	-	-	1	-	1
26	Egypt	-	-	-	-	-	8	-	-	8
27	Cameroon	-	-	-	-	-	1	-	-	1
28	South Africa	-	-	-	-	-	6	8	-	14

First of all MSA was performed for 56 sequences from genotype 3 subtype 3a. After that single MSA was done for all the 346 sequences. Then MSA was performed for 73 sequences from genotype 1 subtype 1b and 3 sequences from genotype 1 subtype 1c. Furthermore, MSA was performed for the 73 sequences from genotype 1 subtype 1b with 64 sequences from genotype 1 subtype 1a, 35 sequences from genotype 5, 37 sequences from genotype 4, 58 sequences from genotype 3 and 58 sequences from genotype 2 respectively. Finally a single phylogenetic tree was constructed for all the 346 sequences using UPGMA method using CLC sequence software http://www.clcbio.com/index.php?id=28.

## Results

### NS4A protein HCV genotype 3a

**Figure 1:**
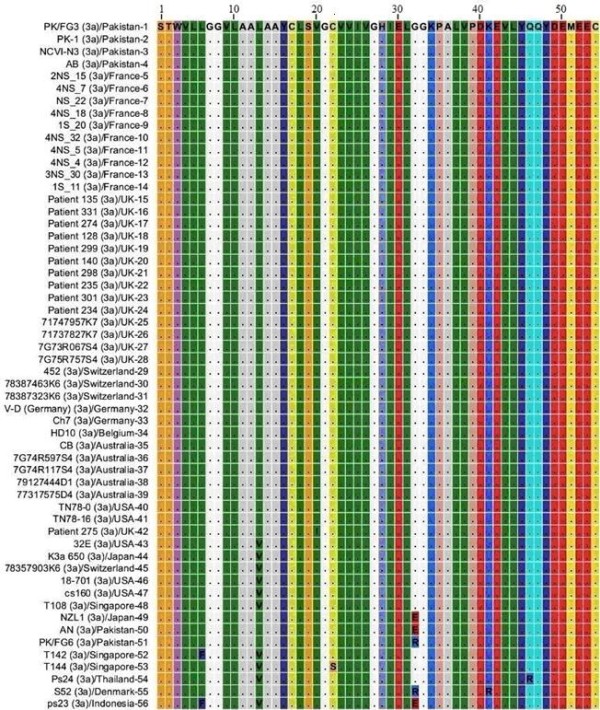
**Multiple Sequence Alignment of NS4A protein of HCV genotype 3a**. Numerical numbers at the top of the figure indicate position of the different amino acids in the NS4A protein. The isolate (genotype)/country-serial number of the sequences are shown at the left side of the figure. Dots and Roman letters in figure indicate similarity and amino acid substitutions respectively relative to the first sequence PK/FG3 taken as a reference sequence.

Total of 56 different amino acid sequences that were reported from different parts of the world for NS4A protein genotype 3 subtype 3a were analyzed through Multiple Sequence Alignment. Out of 56 sequences that were observed 41 sequences had same amino acid sequence as shown in Figure 1, where dots show similarity and Roman letters shows amino acid substitutions relative to the sequence 1 (PK/FG3). PK/FG3 isolate used as a reference sequence was isolated from local Pakistani population. These 41 sequences which show same amino acid sequence for NS4A protein of HCV genotype 3a have been reported from different parts of the world i.e. Pakistan, France, United Kingdom, Switzerland, Germany, Belgium, Australia and United States of America, representing 4 different continents of the world i.e. Asia, Europe, Australia and North America. Different amino acid substitutions F_6_, V_13_, I_20_, S_22_, E_32_, R_32_, R_41 _and R_46 _were observed in sequences 42-56 relative to sequence 1. These results indicate relatively conserved nature of NS4A protein at genotype level and may help in performing evolutionary studies with HCV.

### Amino Acid sequence comparison of NS4A protein of different HCV genotypes

Multiple Sequence Alignment of NS4A protein of HCV genotype 3a provided useful information about its conserved nature. These results indicated that both the conserved nature and occasional amino acid substitution in the NS4A protein might provide useful information about origin of HCV in humans. So we compared amino acid composition of NS4A protein of different HCV genotypes through Multiple Sequence Alignment. Single MSA was performed for all 346 sequences included in this study (data not shown) and amino acid substitutions were critically analyzed in all HCV genotypes. We observed amino acid substitutions in genotype 1b that were consistent members of NS4A protein of different HCV genotypes. So we analyzed and compared sequences of genotype 1b with sequences from different HCV genotypes and subtypes.

### NS4A protein HCV genotype 1b and 1c

**Figure 2:**
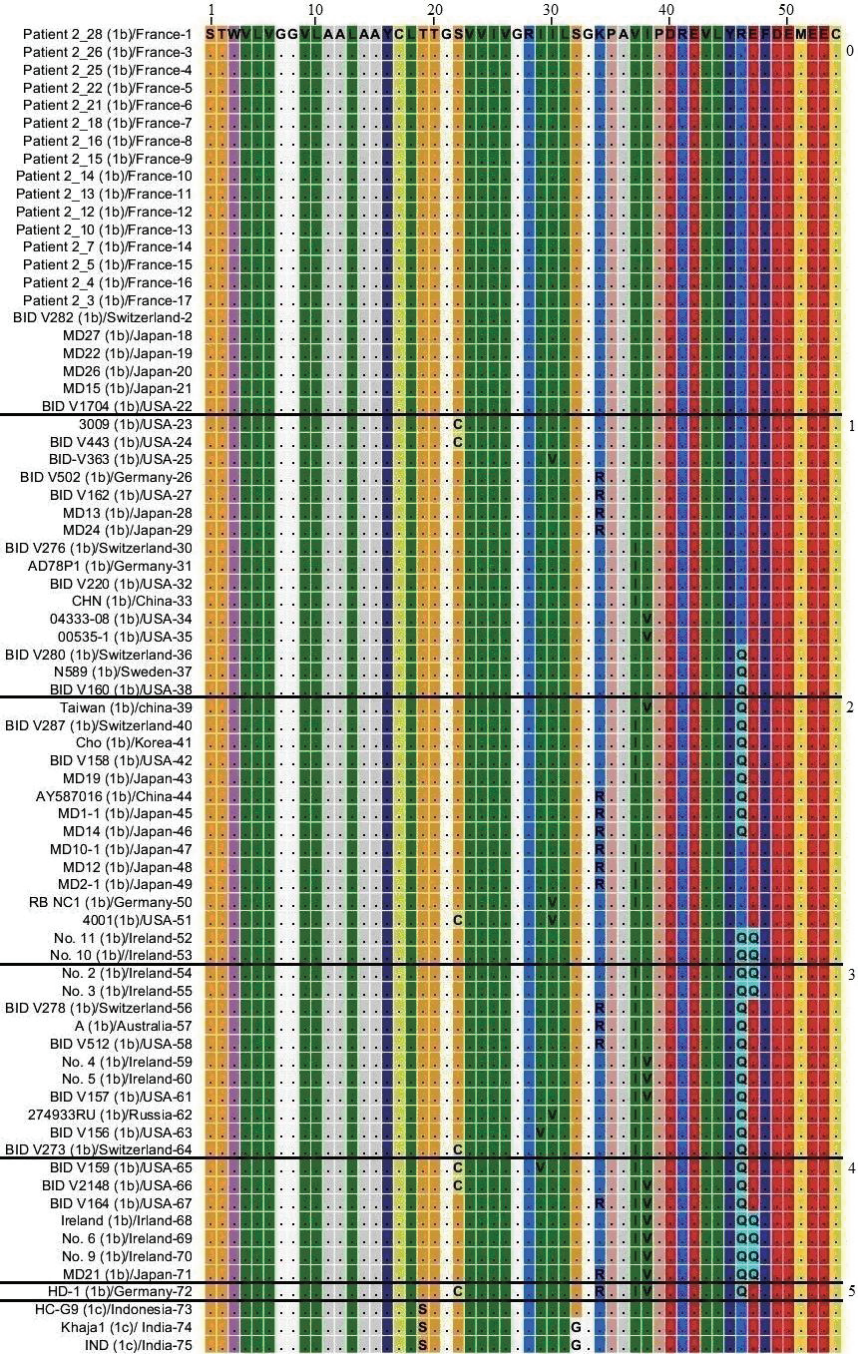
**Multiple Sequence Alignment of NS4A protein of HCV genotype 1b and 1c**. Position of different amino acid is represented by numerical numbers at the top of the figure. The isolate (genotype)/country-serial number of the sequences are shown at the left side of the figure. Dots and Roman letters in figure indicate similarity and amino acid substitutions respectively relative to the first sequence patient 2_28 reported from France. Numerical number on the right side of the figure indicates number of amino acid substitutions in individual sequences.

A total of 72 sequences for NS4A protein HCV genotype 1 subtype 1b and 3 sequences for subtype 1c were compared through Multiple Sequence Alignment as shown in the Figure 2. Genotype 1b sequences included in this study were reported from France, Switzerland, United States of America, Japan, Germany, China, Sweden, Korea, Ireland, Australia and Russia while genotype 1c sequences were reported from Indonesia and India. Sequences 1 to 22 have same amino acid sequence with no amino acid substitution. These 22 sequences were reported from France, Switzerland, Japan and USA, indicating the relatively conserved nature of NS4A protein.

Sequences 23 to 38 have 6 different single amino acid substitutions C_22_, V_30_, R_34_, I37, V_38 _and Q_46 _(Roman letters and numbers indicate specific amino acids and their position in the NS4A protein respectively). Sequence 39 to 51 shows double amino acid substitutions in which the already observed 6 different single amino acid substitutions were combined in pairs and in different combinations. In sequences 52 and 53 another amino acid substitution Q_47 _was found coupled with the already observed substitution Q_46_. Sequences 54 to 64 have three amino acid substitutions in each sequence where the already observed substitutions were found in different combinations except for a new substitution V_29 _in sequence 63. Sequences 65 to 71 have four different amino acid substitutions in each sequence while sequence 72 has five different substitutions C_22_, R_34_, I_37_, V_38 _and Q_46_. So the overall concept we get here is that 6 different kinds of single amino acids substitution that were found from sequences 23 to 38 were somehow combined in different combinations while on the other hand further amino acid substitutions like Q_47 _and V_29 _were introduced as the NS4A protein of genotype 1 subtype 1b evolved.

NS4A protein of genotype 1 subtype 1c closely resembles the NS4A protein of subtype 1b as shown in Figure 2. Sequence 74 shows that NS4A protein of genotype 1 subtype 1c evolved when T_19 _in NS4A protein genotype 1 subtype 1b was substituted to S_19_. G_32 _is another amino acid that we observed in subtype 1c sequences 74 and 75 but not in any of the 72 sequences of the subtype 1b.

### NS4A protein HCV genotype 1a

**Figure 3:**
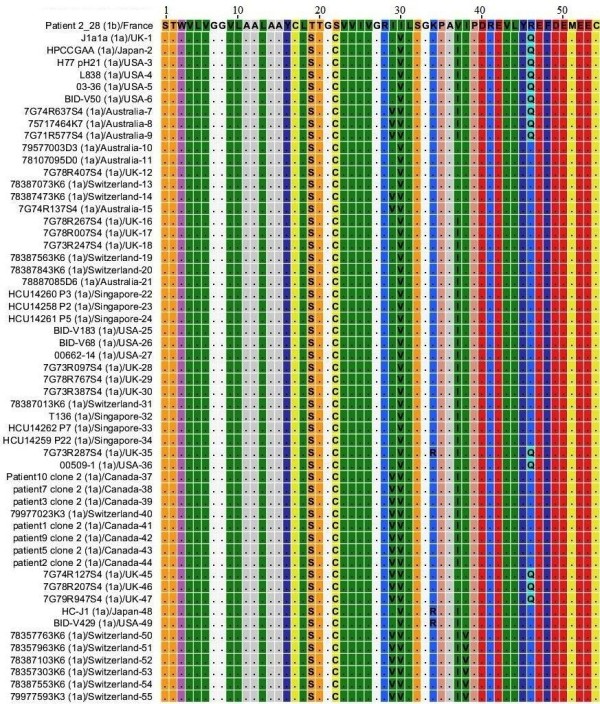
**Multiple Sequence Alignment of NS4A protein of HCV genotype 1b and 1a**. Numerical numbers at the top of the figure indicate position of the different amino acids in the NS4A protein which comprised of total of 54 amino acids. The isolate (genotype)/country-serial number of the sequences are shown at the left side of the figure. Dots and Roman letters in figure indicate similarity and amino acid substitutions respectively relative to the first sequence patient 2_28 reported from France for genotype 1b.

MSA was performed for 64 different sequences of NS4A protein genotype 1 subtype 1a with 72 sequences from genotype 1 subtype 1b and the file that was generated is shown in Figure 3, for convenience only one sequence for genotype 1b is shown. Genotype 1a sequences that are included in this study were reported from France, UK, Japan, USA, Australia, Switzerland, Singapore and Canada. We observed that C_22 _and V_30 _that were introduced as occasional amino acid substitutions in NS4A protein of genotype1b are consistent members of NS4A protein of genotype 1 subtype 1a. R_34_, I_37_, V_38 _and Q_46 _that emerged as single amino acid substitutions in NS4A protein of genotype 1b are also present in different sequences of genotype 1a. S_19 _amino acid which was also observed in genotype 1c sequences is a consistent member of genotype 1a NS4A protein. The overall similarity represented in the form of dots, the presence of C_22 _and V_30 _as consistent members, the presence of V_29_, R_34_, I_37_, V_38 _and Q_46 _amino acids which originally emerged at genotype 1b level clearly indicates that NS4A protein of genotype 1a evolved later as compared to NS4A protein of genotype 1b.

### NS4A protein HCV genotype 5

**Figure 4:**
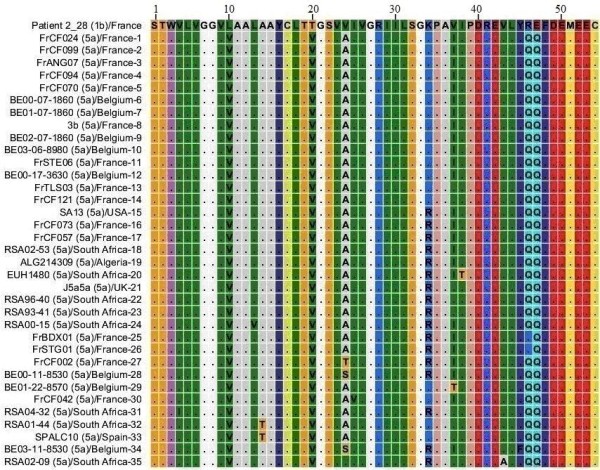
**Multiple Sequence Alignment of NS4A protein sequences of HCV genotype 1b and 5**. Numerical numbers at the top of the figure indicate position of the different amino acids in the NS4A protein which is comprised of total of 54 amino acids. The isolate (genotype)/country-serial number of the sequences are shown at the left side of the figure. Dots and Roman letters in figure indicate similarity and amino acid substitutions respectively relative to the first sequence patient 2_28 reported from France for genotype 1b.

MSA for 35 different sequences of NS4A protein of genotype 5 was performed with 72 sequences form genotype 1 subtype 1b. Genotype 5 sequences that are included in this study were reported from France, Belgium, USA, South Africa, Algeria, UK and Spain. MSA results for genotype 5 sequences are shown in Figure 4 and for simplicity only one sequence from genotype 1b is shown. Comparative analysis of genotype 1b and genotype 5 sequences (Figure 4) shows that L_10_, T_20 _and V_24 _of NS4A protein genotype 1b has been replaced by V_10_, V_20 _and A_24 _respectively in NS4A protein of genotype 5. Q_46 _and Q_47 _are the amino acids that were introduced as amino acid substitutions in genotype 1b sequences has been retained as more consistent members in genotype 5 sequences. R_34 _and I_37 _amino acids are also present in different sequences of genotype 1b and 5. We propose that NS4A protein of genotype 5 evolved when V_10_, V_20 _and A_24 _amino acid substitutions were introduced into NS4A protein sequences of genotype 1b (sequences 52 to 58 in Figure 2).

### NS4A protein HCV genotype 4

**Figure 5:**
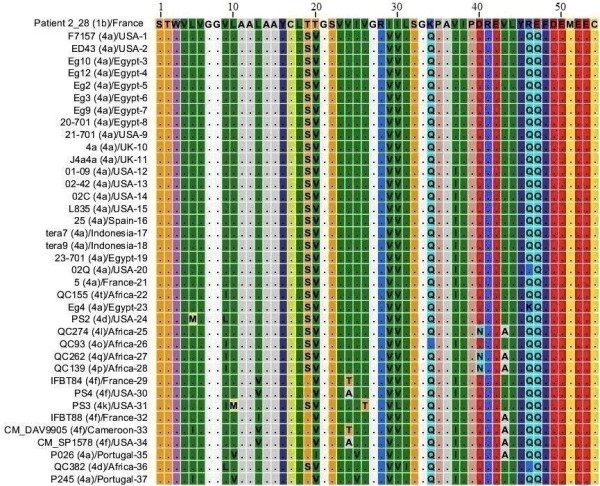
**Multiple Sequence Alignment of NS4A protein sequences of HCV genotype 1b and 4**. Numerical numbers at the top of the figure indicate position of the different amino acids in the NS4A protein which is comprised of total of 54 amino acids. The isolate (genotype)/country-serial number of the sequences are shown at the left side of the figure. Dots and Roman letters in figure indicate similarity and amino acid substitutions respectively relative to the first sequence patient 2_28 reported from France for genotype 1b.

MSA was performed for 37 different sequences of NS4A protein genotype 4 with 72 sequences form genotype 1 subtype 1b. Genotype 4 sequences included in this study were reported from USA, Egypt, UK, Spain, France, Indonesia, Cameroon and Portugal. Some of the sequences for genotype 1b that were reported from African patients in Canada are also included in this study. MSA results are shown in Figure 5 and for simplicity only one sequence from genotype 1b is shown.V_29_, V_30_, Q_46 _and Q_47 _amino acids that emerged as amino acid substitutions in NS4A protein sequences of genotype 1b can be seen to be present more consistently in NS4A protein of genotype 4. I_37 _amino acid can also be seen in some sequences. Q_34 _amino acid has been observed to be present consistently in NS4A protein sequences of genotype 4 only. S_19 _and V_20 _are the other amino acids that are present more consistently in NS4A protein sequences of genotype 4 but not in the sequences that we had observed for genotype 1b. Other amino acids occurring less frequently are also shown in Figure 5.

### NS4A protein HCV genotype 6

**Figure 6:**
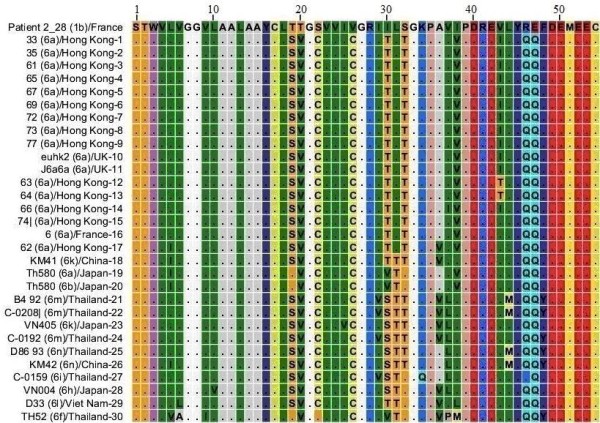
**Multiple Sequence Alignment of NS4A protein sequences of HCV genotype 1b and genotype 6**. Numerical numbers at the top of the figure indicate position of the different amino acids in the NS4A protein which is comprised of total of 54 amino acids. The isolate (genotype)/country-serial number of the sequences are shown at the left side of the figure. Dots and Roman letters in figure indicate similarity and amino acid substitutions respectively relative to the first sequence patient 2_28 reported from France for genotype 1b.

Thirty amino acid sequences for NS4A protein genotype 6 were uploaded to the CLC software and MSA was performed with 72 sequences from genotype 1 subtype 1b. Genotype 6 sequences that were included in this study were reported from Hong Kong, UK, France, China, Japan, Thailand and Viet Nam. Results for this alignment are shown in Figure 6, for convenience only one sequence from genotype 1b is shown. It is clear from the figure that C_22_, Q_46 _and Q_47 _are present as more consistent members of NS4A protein sequences of genotype 6. These amino acids emerged as amino acid substitutions in NS4A protein of genotype 1b. V_38 _amino acid present in different sequences of genotype 6 also emerged in genotype 1b sequences. S_19_, V_20_, C_26_, T_30_, T_31_, T_32_, I_43 _are the amino acids that are present in different sequences of genotype 6 but not in the 72 sequences we observed for genotype 1b. Some other amino acids shown in Figure 6 are also present in genotype 6 sequences but they occur less consistently.

### NS4A protein HCV genotype 3

**Figure 7:**
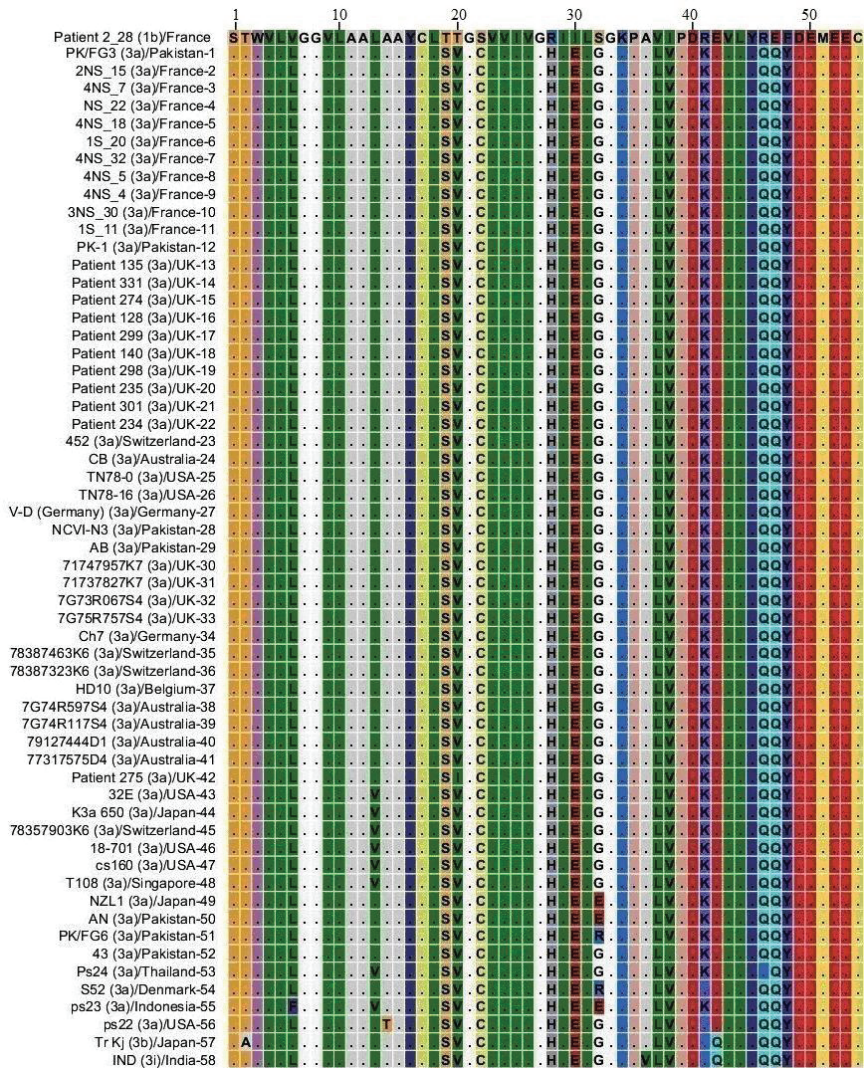
**Multiple Sequence Alignment of NS4A protein sequences of HCV genotype 1b and 3**. Numerical numbers at the top of the figure indicate position of the different amino acids in the NS4A protein which is comprised of total of 54 amino acids. The isolate (genotype)/country-serial number of the sequences are shown at the left side of the figure. Dots and Roman letters in figure indicate similarity and amino acid substitutions respectively relative to the first sequence patient 2_28 reported from France for genotype 1b.

MSA was performed for 58 sequences of NS4A protein of genotype 3 and 72 sequences from genotype 1b. Genotype 3 sequences included in this study were reported from Pakistan, France, UK, Switzerland, Australia, USA, Germany, Belgium, Japan, Singapore, Denmark, Indonesia and India. Results for this alignment are shown in Figure 7, for convenience only one sequence for genotype 1b is shown. C_22_, V_38_, Q_46 _and Q_47 _amino acids are frequent members of NS4A protein sequences of genotype 3. These amino acids emerged as amino acid substitutions in NS4A protein sequences of genotype 1b. Presence of S_19 _and G_32 _amino acids together in same sequence has been observed in sequences from genotype 3 and 1c only. L_6_, V_20_, H_28_, E_30_, L_37_, K_41 _and Y_48 _are amino acids that we did not observe in our sequences for genotype 1b but are frequent members of NS4A protein sequences from genotype 3. Some other amino acids differences have also been observed but are present less frequently as shown in Figure 7.

### NS4A protein HCV genotype 2

**Figure 8:**
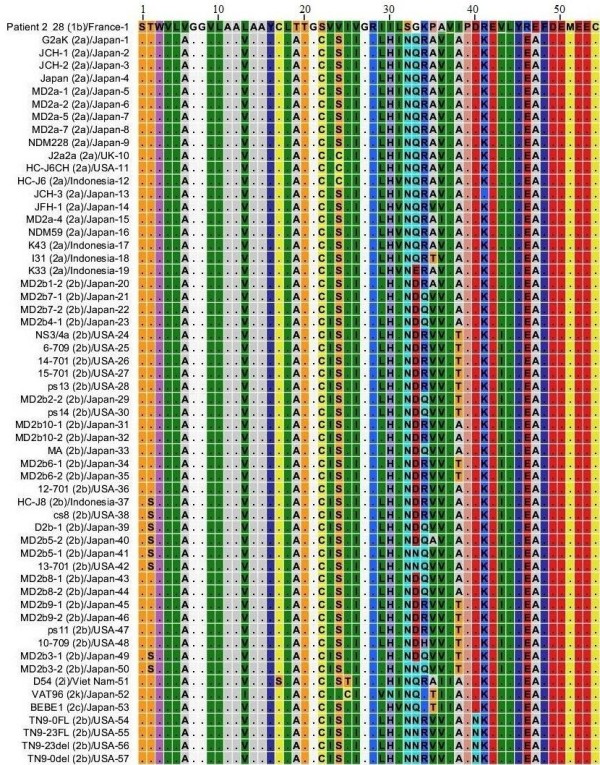
**Multiple Sequence Alignment of NS4A protein sequences of HCV genotype 1b and 2**. Numerical numbers at the top of the figure indicate position of the different amino acids in the NS4A protein which is comprised of total of 54 amino acids. The isolate (genotype)/country-serial number of the sequences are shown at the left side of the figure. Dots and Roman letters in figure indicate similarity and amino acid substitutions respectively relative to the first sequence patient 2_28 reported from France for genotype 1b.

58 sequences for NS4A protein genotype 2 that were reported from Japan, UK, USA, Indonesia, and Viet Nam were included in this study. MSA was performed for 58 sequences from genotype 2 and 72 sequences from genotype 1b for NS4A protein. Results are shown in Figure 8, for convenience only one sequence for genotype 1b is shown. C_22 _is the amino acid that appeared as occasional substitution in NS4A protein of genotype 1b but is more frequent member of NS4A protein sequences from genotype 2. K_41 _is a frequent member of genotype 2 and genotype 3 sequences. NS4A protein sequences from genotype 2 differs the most from genotype 1b sequences in terms of amino acid composition as indicated in Figure 8.

### Phylogenetic Analysis

**Figure 9:**
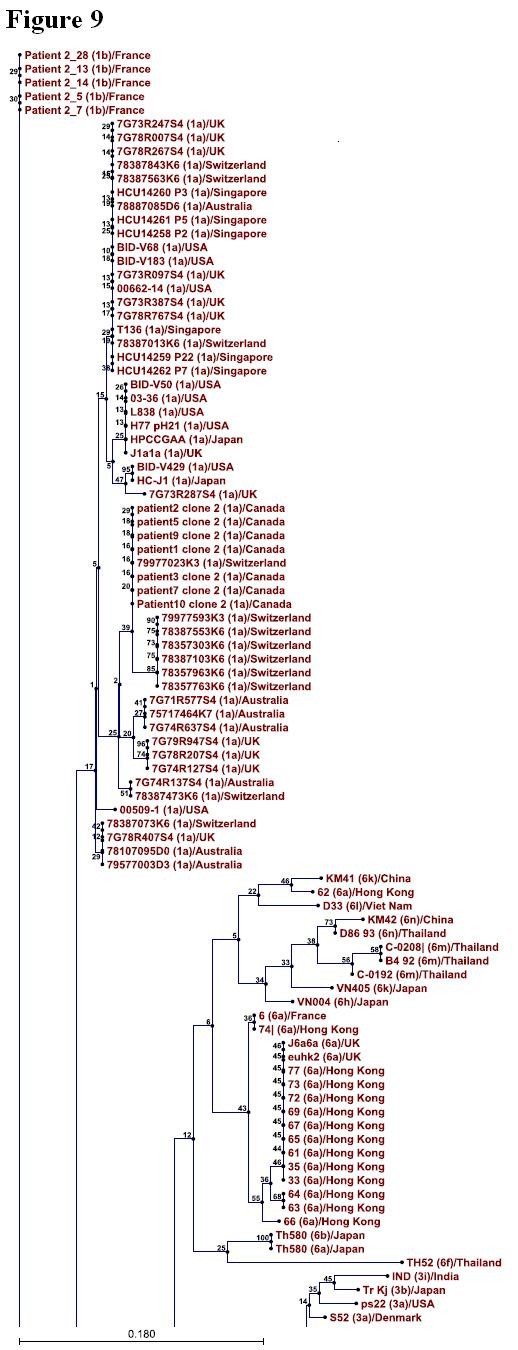
**Phylogenetic tree constructed for 357 sequences from 6 so far known HCV genotypes using CLC sequence viewer software and UPGMA method**. Default value of 100 was used for bootstrap analysis and corresponding values are shown on the individual branches. For convenience, Phylogenetic tree is divided into four figures 9-12. These figures should be considered in continuation. Figure 9 is showing sequences of genotype 1b at the root while clustering 1a, 6 and some sequences from genotype 3.

**Figure 10:**
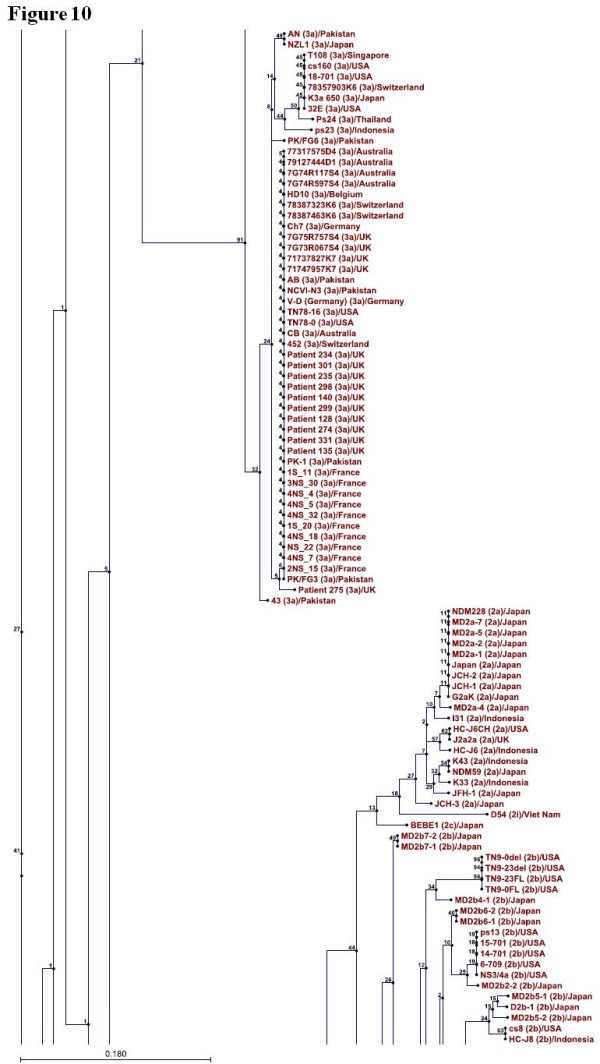
**Continuation of figure 9**. Figure 10 is showing clustering of the genotype 3 and 2 projecting away from the root of the tree.

**Figure 11:**
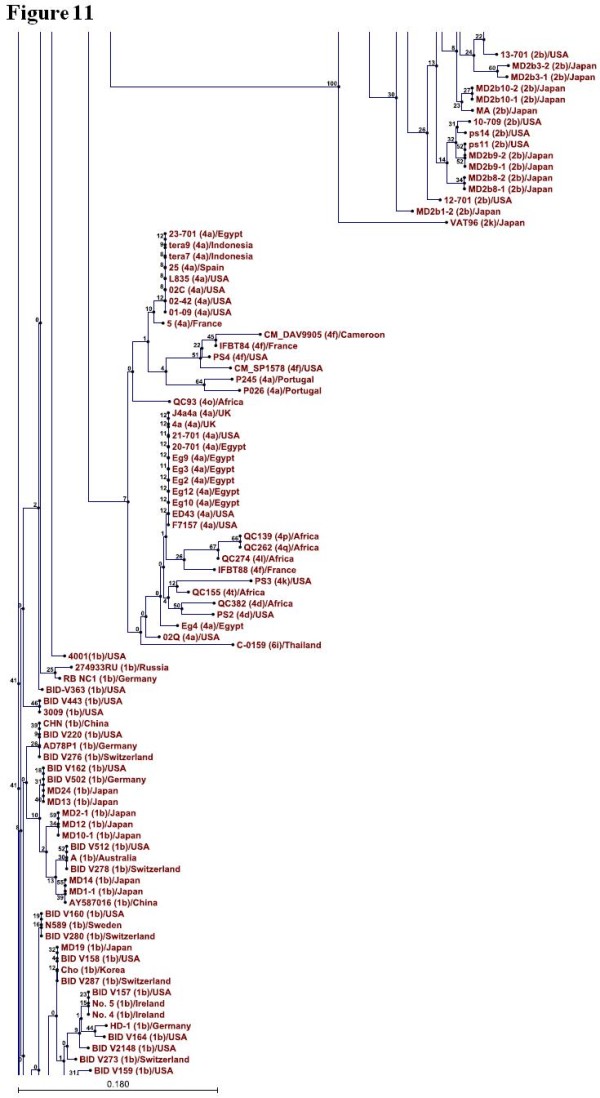
**Continuation of figure 10**. Figure 11 is showing clustering of the genotype 2 and 4 projecting away from the root of the tree while genotype 1 sequences near root of the tree.

**Figure 12:**
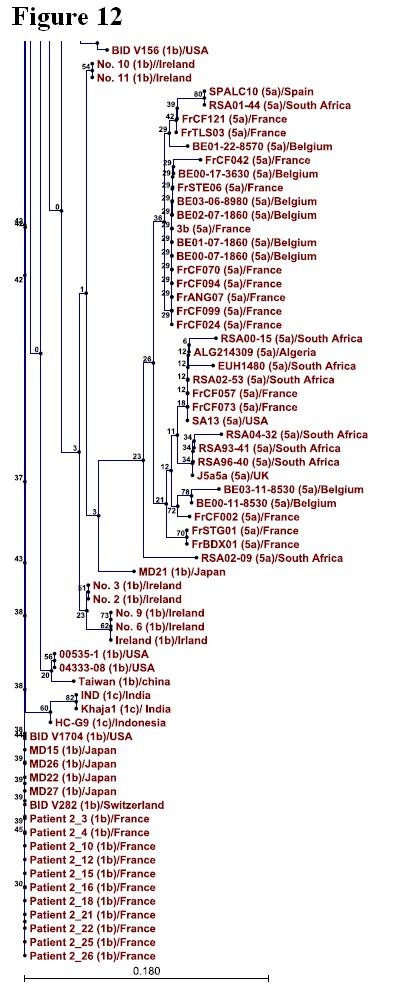
**Phylogenetic tree showing sequences of genotype 1b at or near the root of the Phylogenetic tree while clustering of genotype 5 sequences projecting away from the root**. Figure 12 is in continuation of figure 11.

Phylogenetic tree was constructed for 346 sequences of NS4A protein representing so far known HCV genotypes using CLC sequence viewer software and through UPGMA method. Standard layout of the tree is shown in Figure 9, 10, 11, 12 (A single Phylogenetic tree was constructed but for convenience it has been shown in four different figures and these figures should be considered in continuation from Figure 9, 10, 11, 12). UPGMA method assumes that evolution has occurred at a constant rate in the different lineages and that is why root of the tree can also be estimated. For bootstrap analysis the default value of 100 was used. Bootstrap values are attached to each branch. Genotype 1b sequences occupy the root of the tree and sequences from the individual genotypes are clustered together in the tree which clearly demonstrates that NS4A protein of different HCV genotypes originally evolved from NS4A protein of genotype 1b.

## Discussion

NS4A gene (Accession no. HM135518 and isolate name PK/FG3) that we had isolated, sequenced and reported to the Gen Bank from a Pakistani patient chronically infected with HCV genotype 3a showed 100% homology on protein blast available at NCBI with many sequences reported from United Kingdom. This was an amazing observation as HCV is known for a high mutation rate but still NS4A protein reported from Pakistani and UK populations show such a high similarity at amino acid level. These Blast results prompted us to investigate the conserved nature of NS4A protein across different regions of the world.

Our results in Figure 1 clearly shows that Hepatitis C virus genotype 3a is widespread to the four different continents of the world but it still retained same amino acid sequence for NS4A protein despite high mutation rate in HCV genome. The relatively conserved nature of NS4A protein indicates that the original NS4A protein, which was part of HCV polyprotein when it first established itself in humans, might have been passed on in its dormant form to the present day HCV and its sequence might have been reported to sequence databases. And by comparing the amino acid composition of NS4A protein of different HCV genotypes, the occasional amino acid substitutions that we had observed might help us to investigate its identity.

The conserved nature of NS4A protein has two important implications. First when amino acid substitutions are introduced into this protein, there is a considerable chance that they will be retained in future progenies. And secondly, some of these amino acid substitutions may travel a long distance across different HCV genotypes. By locating such amino acid substitutions and following them across different HCV genotypes, might help us identify the genotypes that evolved earlier or later in HCV evolution. Our study suggests that C_22_, Q_46 _and Q_47 _are three very important amino acid substitutions that were introduced into NS4A protein of genotype 1b early in HCV evolution. Amino acid composition analysis of NS4A protein of different HCV genotypes shows that at least one of the three amino acids is a consistent member of NS4A of the all other known HCV genotypes. C_22 _is a more consistent member of NS4A protein sequences of genotype 1a, genotype 6, genotype 3 and genotype 2. Q_46 _and Q_47 _amino acids are more consistent members of NS4A protein sequences of genotype 5, genotype 4, genotype 6 and genotype 3. V_29_, V_30 _and V_38 _are the other three important amino acid substitutions introduced into NS4A protein of genotype 1b. V_30 _is a consistent member of NS4A protein sequences of genotype 1a, V_29 _and V_30 _are more consistent members of genotype 4 sequences and V_38 _is more consistent member of genotype 3 sequences.

Previous studies that were performed to understand HCV evolution and to classify different genotypes used nucleotide sequences [5,6,19,20]. We have used amino acid sequences in this study because sequence divergence is very high in HCV at nucleotide level due to error-prone nature of its polymerase. For the study of evolutionary history and origin of new subtypes of HCV there is a need of consistent system. We used amino acid substitution in individual genotypes and subtypes of HCV for the study of origin and evolution. Suzuki and Nei used amino acid sequences to study the origin and evolution of Influenza virus [7]. Furthermore previous studies used 5UTR, Core/E1 and or NS5B gene regions [6,19,21,22]. While on the other hand we have used relatively conserved NS4A protein sequences which can better predict the picture of evolution. Previous studies used ClustalW for Multiple Sequence Alignment, we have used CLC software that automatically arranges sequences on the basis of sequence similarity. Furthermore, CLC software allows the movement of individual sequences up and down in the MSA file that is generated. So we can arrange sequences in different orders and look for different patterns of amino acid substitutions that may emerge.

We have identified different amino acids as consistent members in different HCV genotypes that we did not observed in our NS4A protein sequences from genotype 1b. We believe that these amino acids were introduced later as HCV evolved with time. T_19 _and S_32 _amino acids in genotype 1b sequences have been replaced by S_19 _and G_32 _in genotype 1c sequences respectively. T_19 _of genotype 1b sequences has been replaced by S_19 _in genotype 1a sequences. L_10_, T_20 _and V_24 _in genotype 1b sequences have been replaced by V_10_, V_20 _and A_24 _in genotype 5 sequences respectively. Genotype 4 sequences have S_19_, V_20 _and Q_34 _amino acids as more consistent members while genotype 1b sequences have T_19_, S_32 _and K_34 _amino acids. Genotype 6 and genotype 3 sequences also have S_19 _and V_20 _amino acids similar to genotype 4 sequences. T_30 _and T_32 _are also members of genotype 6 sequences but these are less consistent members compared to S_19 _and V_20 _amino acids. R_28_, I_30_, S_32_, V_37_, K_41_, F_48 _in genotype 1b sequences has been replaced by H_28_, E_30_, G_32_, L_37_, K_41_, Y_48 _in genotype 3 sequences. Genotype 2 shows highest diversity from genotype 1b sequences in terms of amino acid composition and is indicated in Figure 8. The overall similarity of genotype 1b sequences with other genotypes denoted by dots (Figure 2 to Figure 8), the occasional amino acid substitutions in genotype 1b and their presence as more consistent members in sequences of other known genotypes and presence of further substitutions that we just discussed shows that NS4A protein of the other so far known HCV genotypes originally evolved from NS4A protein of genotype 1b.

To further confirm our results phylogenetic analysis was performed by constructing a single phylogenetic tree using UPGMA method as shown in Figure 9, 10, 11, 12. Many studies related to HCV classification and evolution has used UPGMA method for constructing phylogenetic tree [23-25]. NS4A protein sequences from genotype 1b occupied the root of the phylogenetic tree. Sequences from individual genotypes were clustered together in the tree which indicates that our constructed tree is in accordance with current classification system which is based on nucleotide sequence analysis of 5TUR, Core/E1 and NS5B gene regions. This also shows the importance of NS4A protein as a phylogenetic marker of HCV history and UPGMA as a relevant method for tree construction. Both amino acid composition analysis and our phylogenetic tree indicates that genotype 2 differ the most from genotype 1b than any other HCV genotype. Based on the above mentioned observations it is now easy to generalize that HCV genotype 1b established itself earlier in humans and that all other known HCV genotypes evolve later as result of mutations in genotype 1b. We propose that the following amino acid sequence (Figure 2, Sequence 1 to 22) might have been sequence of the NS4A protein which was part of HCV polyprotein when it first infected humans.

S T W V L V G G V L A A L A A Y C L T T G S V V I V G R I I L S G K P A V I P D R E V L Y R E F D E M E E C

Some of the genotype 6 variants reported from Southeast Asia have 5'UTR sequences identical to those of genotype 1b and 1a [26-29]. At nucleotide level, 5'UTR is the most conserved region in HCV genome and these reports support our results. Few of the HCV genomic sequences reported from Russia have structural genes similar to genotype 2 and non-structural genes similar to genotype 1b [30,31], which according to our findings is the parent HCV genotype. Another genomic sequence reported from Peru has structural genes similar to genotype 1a and non-structural genes similar to genotype 1b [32]. These sequences have been classified as recombinants because it is believed that these sequences were generated as a result of recombination events between different HCV genotypes [30-32]. It is well documented that HCV target structural genes like E1 and E2 for mutation to avoid immune responses [33,34]. There is a possibility that these recombinant genotypes evolved as result of much higher mutation rate than normal in the structural region and lower mutation rate in non-structural regions and not as a result of recombination events. This much higher mutation rate could be due to high pressure on HCV from immune system in certain individuals. But much work needs to be done to establish facts regarding recombinants genotypes and our discovery will have a role to play in that regard.

## Conclusion

This work highlights the significance of NS4A protein as phylogenetic marker in studies related to origin and evolution of HCV. Amino acid substitution and phylogenetic analysis of NS4A protein sequences of different HCV genotypes shows that NS4A protein of the so far known HCV genotypes evolved from NS4A protein of HCV genotype 1b. This implies that genotype 1b established itself earlier in humans and that all other known HCV genotypes evolved later as a result of mutations in HCV genotype 1b.

## Abbreviations

HCV: hepatitis C;

## Competing interests

The authors declare that they have no competing interests.

## Authors' contributions

MTS, BI, WA and SH designed the study and wrote paper. AS, MA, UAA, SG, SA, MI and IS analyzed and arranged the data. All work was performed under supervision of SH. All authors read and approved the final manuscript.

## Authors' information

Bushra Ijaz (M Phil Molecular Biology), Waqar Ahmad (M Phil Chemistry), and Sana Gull (MSc Biochemistry) are Research Officer at CEMB. Aleena Samrin and Usman Ali Ashfaq are PhD in Molecular biology, while Muhammad T Sarwar, Muhammad Ansar, Humera Kausar, Sultan Asad and Imran Shahid are PhD scholars. Sajida Hassan (PhD Molecular Biology) is principal investigator at CEMB, University of the Punjab, Lahore.
